# Low Seroprevalence of SARS-CoV-2 among Healthcare Workers in Malaysia during the Third COVID-19 Wave: Prospective Study with Literature Survey on Infection Prevention and Control Measures

**DOI:** 10.3390/healthcare10101810

**Published:** 2022-09-20

**Authors:** Nik Mohd Noor Nik Zuraina, Mohd Zulkifli Salleh, Mohd Habil Kamaruzaman, Nur Suhaila Idris, Alwi Muhd Besari, Wan Mohd Zahiruddin Wan Mohammad, Nabilah Ismail, Ahmad Sukari Halim, Zakuan Zainy Deris

**Affiliations:** 1 Department of Medical Microbiology & Parasitology, School of Medical Sciences, Universiti Sains Malaysia Health Campus, Kota Bharu 16150, Malaysia; 2 Department of Family Medicine, School of Medical Sciences, Universiti Sains Malaysia Health Campus, Kota Bharu 16150, Malaysia; 3 Hospital Universiti Sains Malaysia, Universiti Sains Malaysia Health Campus, Kota Bharu 16150, Malaysia; 4 Department of Medicine, School of Medical Sciences, Universiti Sains Malaysia Health Campus, Kota Bharu 16150, Malaysia; 5 Department of Community Medicine, School of Medical Sciences, Universiti Sains Malaysia Health Campus, Kota Bharu 16150, Malaysia; 6 Reconstructive Sciences Unit, School of Medical Sciences, Universiti Sains Malaysia Health Campus, Kota Bharu 16150, Malaysia

**Keywords:** seroprevalence, SARS-CoV-2, COVID-19, healthcare workers, infection prevention and control measures, movement control order

## Abstract

Healthcare workers (HCWs) are at greater risk for severe acute respiratory syndrome coronavirus-2 (SARS-CoV-2) infection. This serology surveillance study aimed to investigate the prevalence of SARS-CoV-2 antibodies among the HCWs who were asymptomatic during the third wave of COVID-19 in Malaysia. HCWs from the Universiti Sains Malaysia (USM) Health Campus were prospectively recruited between August 2020 and March 2021 on a voluntary basis. Data on socio-demographics, possible risk factors and travel history were recorded. Serological diagnoses from serum samples were examined for total antibodies against SARS-CoV-2 using an immunoassay kit. A literature survey was performed on the compliance with infection and prevention control (IPC) practices for COVID-19 among HCWs. The majority of the total 617 HCWs participating in this study were nurses (64.3%, *n* = 397), followed by health attendants (20.9%, *n* = 129), medical doctors (9.6%, *n* = 59) and others (6.3%, *n* = 39). Of those, 28.2% (*n* = 174) claimed to have exposure to COVID-19 cases, including history of close contact and casual contact with infected patients. Most importantly, all serum samples were found to be non-reactive to SARS-CoV-2, although nearly half (40.0%, *n* = 246) of the HCWs had been involved directly in the management of acute respiratory illness cases. A proportion of 12.7% (*n* = 78) of the HCWs reported having underlying health problems, such as diabetes mellitus, hypertension and hyperlipidemia. Despite the presence of medical and sociological risks associated with SARS-CoV-2 infections, the current study found zero prevalence of antibodies against SARS-CoV-2 among the HCWs of USM. Based on the literature survey, the vast majority of Malaysian HCWs demonstrated good IPC practices during the pandemic (average percentage ranged between 92.2% and 99.8%). High compliance with IPC measures may have led to the low seroprevalence of SARS-CoV-2 among the HCWs.

## 1. Introduction

Coronavirus disease 2019 (COVID-19), which is caused by severe acute respiratory syndrome coronavirus 2 (SARS-CoV-2), is currently underway and has resulted in significant morbidity and mortality worldwide. It is a global phenomenon that has caused substantial economic, social and political challenges, leading to lockdowns and travel restrictions, as well as severely disrupting public health services. As of September 2022, accumulative confirmed cases from all over the world have reached the alarming total of 613 million, with more than 6.5 million deaths. The beta-coronavirus SARS-CoV-2 typically induces respiratory-like illnesses that potentially progress into pneumonia, with mild to severe symptoms, including fever or chills, breathing difficulties, cough, sore throat, headache, nausea, anosmia (loss of smell) or ageusia (loss of taste) and diarrhea [1]. Viruses naturally mutate over time. Mutations induce alterations in the spike (S) glycoprotein protein structures that change its antigenic properties and can potentially reduce neutralization by neutralizing antibodies [2]. Variants such as Alpha (lineage B.1.1.7), Beta (lineage B.1.351), Gamma (lineage P.1) and Delta (lineage B.1.617.2) are concerning due to their potential immune evasion, as well as their enhanced transmissibility and infectivity [3,4,5]. These variants could potentially limit vaccine effectiveness; thus, achieving herd immunity with vaccinations could be more difficult [6].

Malaysia documented its first COVID-19 cases in January 2020 involving three tourists from China [7]. As of September 2022, the total confirmed cases of COVID-19 infection in Malaysia have reached 4.8 million, with more than 36.2 thousand coronavirus-related deaths [8]. Malaysia has experienced five waves of the COVID-19 outbreak. The first wave (25 January 2020–16 February 2020) accumulated 22 cases. During the second wave (27 February 2020–30 June 2020), several local clusters emerged and led to a massive spike of cases all over the country. This brought Malaysia to the first enforcement of a Movement Control Order (MCO), which was effectively started on 18 March 2020 and extended until May 2020. Subsequently, several series of MCOs have been enforced as a mitigation effort to curb the spread of COVID-19 in the country, decreasing daily cases from three-digit figures during the second wave to two- or one-digit figures throughout the MCOs up to August 2020 [8]. The third wave was sparked by several clusters associated with the Sabah state election that took place on 26 September 2020 due to the extensive domestic travelling across the country by politicians, voters and supporters following the announcement of the election on 9 August [9]. Positive COVID-19 cases continually increased to four-digit numbers per day and overburdened the healthcare system. Hence, reimplementation of the MCO was initiated in the middle of January 2021, being lifted in early March 2021 when the cases declined. Relaxation of the MCOs and the resulting increase in communal movement rates has been translated into spikes of COVID-19 cases all over the country. The fourth (April–September 2021) and the fifth (February–March 2022) waves, which hit over 20,000 cases per day, corresponded to the emergence of the Delta and Omicron variants, respectively.

Healthcare workers (HCWs) are crucial for the continuous functioning of health systems during the global COVID-19 pandemic and are unequivocally exposed to a higher risk of infection than the general population through direct contact with COVID-19 patients. Therefore, if infected, they pose a risk to other vulnerable patients, as well as their fellow HCWs. If SARS-CoV-2 transmission rises, the number of HCWs available may become inadequate to respond to the healthcare demand. Hence, in addition to MCOs and the COVID-19 vaccination program, the Malaysian Government, through the Ministry of Health, has issued several infection and prevention control (IPC) measures to prioritize HCWs’ protection against COVID-19 [9,10,11]. All Malaysians, including HCWs, are advised to follow the new norms at the workplace and in public by practicing the “3Ws” (wear a mask, wash hands and heed COVID-19 warnings) and avoiding the “3Cs” (crowded places, confined spaces and closed conversation). Additional IPC measures for HCWs include the mandatory use of appropriate personal protection equipment (PPE) in clinical settings when dealing with patients or specimens, performing effective cleaning and disinfection of high touch areas and maintaining social distancing. In general, Malaysians have demonstrated acceptable levels of knowledge, attitudes and practices in response to the COVID-19 pandemic since the first MCO started [12]. 

Seroprevalence studies provide essential information on the percentage of people in a population who have experienced recent or past infections. To determine the cumulative infection rate in community and population subgroups, such as HCWs, serological antibody assays are crucial. Such assays can be utilized for the detection of exposure to SARS-CoV-2 by measuring antibody titers in human serum and plasma. This is important, as a seroprevalence study can be used to monitor the prevalence of COVID-19 infection among HCWs and, thus, high-risk hospital personnel and departments can be identified so that unnecessary quarantines and wasteful healthcare resource planning can be avoided. Seroprevalence studies in HCWs have demonstrated a wide range of seropositivity levels that differ by region and time [13]. Asia (4%) was reported to have the lowest seroprevalence of SARS-CoV-2 among HCWs compared to North America (12.7%), Europe (8.5%) and Africa (8.2–45.1%) [14,15]. To date, there have been five studies reporting COVID-19 seroprevalence among different populations in Malaysia during the epidemic and pandemic phases of the pre-vaccination period [16,17,18,19,20]. Their study populations comprised HCWs, residual serum samples from a teaching hospital, local and migrant workers from worksites and blood donors, which showed reported seroprevalences of 0.0–4.5%, 0.4%, 12.1–99.9% and 4.06%, respectively. Only one recent study reported post-vaccination seroprevalence among Malaysian HCWs, in which all the vaccinated subjects were successfully seroconverted (*n* = 148) [21]. Our study aimed to estimate the prevalence of antibodies against SARS-CoV-2 in HCWs from the USM Health Campus, which accommodates 723 beds for the Hospital USM, one of the main hospitals in Malaysia for the diagnosis and treatment of COVID-19. This study describes the detailed setup of a seroprevalence assay during the third wave of the COVID-19 pandemic in Malaysia to determine humoral immune response to SARS-CoV-2 in hospital personnel and staff of the USM Health Campus. This study also has added value in the form of surveillance of IPC practices among Malaysian HCWs towards the prevention of COVID-19.

## 2. Materials and Methods

### 2.1. Subject Recruitment and Data Collection

A prospective, cross-sectional study was conducted involving the HCWs of USM. Information about the study procedures was advertised across the hospital facilities and through electronic media. Aiming for 50% as the prevalence of seropositivity among HCWs [16], 5% precision, 95% confidence intervals and a 20% drop-out rate, the minimum number of participants required for this study was calculated as 482. Participants were recruited on a voluntary basis and consented upon participation. USM HCWs who were aged above 18 years old and asymptomatic for influenza-like illness and had not received any COVID-19 vaccination were eligible to join this study. HCWs who had acute respiratory illness symptoms (fever, cough, sore throat, conjunctivitis, shortness of breath, anosmia and myalgia) within 14 days before sampling were excluded from this study. A standardized, self-administered proforma for socio-demographics, underlying health conditions, history of exposure to COVID-19 patients and history of travel to COVID-19 high-risk areas was distributed to the participants. The use of these data to assess seroprevalence in the population was approved by the Institutional Review Board of the Human Research Ethics Committee, USM, Malaysia (USM/JEPeM/COVID19-29). 

### 2.2. Blood Sampling

Blood sampling was conducted from 24 August 2020 to 9 March 2021. Three milliliter of peripheral venous blood was collected in serum separator tubes containing clot activator and gel separator. Blood samples were delivered to the laboratory of Medical Microbiology and Parasitology, USM. Prior to serum collection, the blood was allowed to set at room temperature for 1 h and centrifuged at 1500× *g* for 3 min. Each serum was transferred to a sterile collection tube and stored at −20 °C for further use in antibody testing with the immunoassay method.

### 2.3. SARS-CoV-2 Antibody Testing by Immunoassay

Detection of total antibodies against SARS-CoV-2 in the enrolled HCWs’ serum samples was performed using an Elecsys^®^ Anti-SARS-CoV-2 immunoassay on a Cobas e411 Analyzer (Roche Diagnostics, Basel, Switzerland), following the manufacturer’s instructions. In addition to the positive and negative controls provided in the assay kit, four sera from post-COVID-19 confirmed cases were also included as additional controls to verify the performance of this test. This double-antigen sandwich ELISA assay uses a recombinant protein representing the nucleocapsid (N) antigen, which favors qualitative detection of high-affinity antibodies against SARS-CoV-2. The system provides results as a cutoff index (COI) in numerical values, whereby a COI ≥ 0.8 U/mL shows that the sample is reactive (positive for anti-SARS-CoV-2 antibodies) and COI < 0.8 U/mL indicates non-reactivity (negative for anti-SARS-CoV-2 antibodies). Detection of reactive antibodies against SARS-CoV-2 in individuals with this assay indicated that they may have had adaptive immune response prior to exposure to SARS-CoV-2.

### 2.4. Literature Survey on IPC Measures against COVID-19 in Malaysia

A literature survey on IPC measures against COVID-19 in Malaysia was performed based on the compliance with IPC practices of Malaysian HCWs towards the prevention of COVID-19. Online articles were retrieved from PubMed and ScienceDirect databases using the search terms “infection and prevention control”, “hand hygiene”, “social distancing”, “personal protection equipment” or “knowledge and practice”, combined with “healthcare worker” and “Malaysia”. A total of seven relevant original articles available from the websites were included in the survey. These studies used self-administered questionnaires to assess knowledge, attitudes/perceptions and practices regarding COVID-19 preventive measures based on the WHO assessment protocol for potential COVID-19 risk factors among HCWs in a healthcare setting. 

### 2.5. Data Analysis

All data were recorded into a Microsoft Excel spreadsheet. Microsoft Excel was also used to perform simple descriptive analyses for frequencies, percentages and means. Categorical variables for sociodemographics and potential risks for COVID-19, such as health status and history of exposure to SARS-CoV-2, were presented in frequencies and percentages. Detection of anti-SARS-CoV-2 nucleocapsid total antibodies from HCWs, post-COVID-19 controls and test controls was analyzed as the means of the COI values.

## 3. Results

### 3.1. Sociodemographic Surveillance of HCWs in the USM Health Campus

A total of 617 HCWs from the USM Health Campus participated in this study, with most of the HCWs being female (79.6%, *n* = 491) and aged ≤ 40 years old (78.5%, *n* = 466). The majority were nurses (64.3%, *n* = 397), followed by health attendants (20.9%, *n* = 129), medical doctors (9.6%, *n* = 59) and other professions (6.3%, *n* = 39), including medical lab technologists (*n* = 8), operational assistants (*n* = 5), science officers (*n* = 5), pharmacists (*n* = 2), a radiographer (*n* = 1) and a dietitian (*n* = 1) (Table 1). In terms of the sampling period, 355 (57.5%) of the participants were recruited between August and December 2020, while the rest (262; 42.5%) of the participants were recruited between January and March 2021. The sampling period corresponded to the third COVID-19 wave in Malaysia.

### 3.2. Health Conditions of the Study Participants

**Figure 1:**
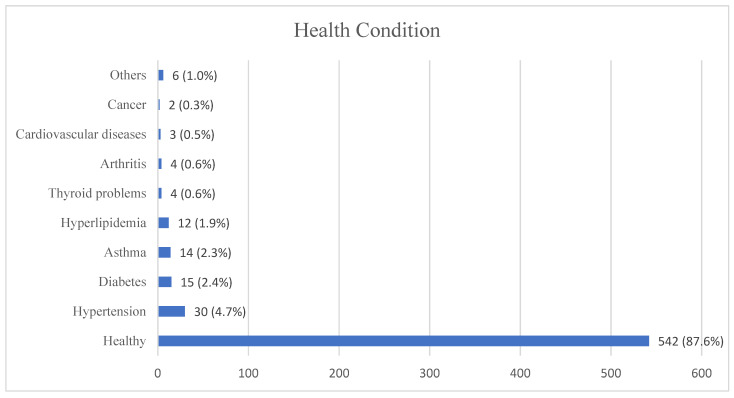
Health conditions of the study participants.

Health conditions were surveyed to address the burden of non-communicable diseases among the HCWs. Underlying chronic medical illness is known to be one of the risk factors for COVID-19. In this study, only 12.4% (*n* = 75) of participants claimed to have underlying diseases, such as hypertension, diabetes mellitus, asthma and hyperlipidemia. A small proportion (1.0%, *n* = 6) reported having health problems in relation to the sinuses and kidneys and gastrointestinal diseases. The vast majority of them were healthy (Figure 1).

### 3.3. History of Exposure to SARS-CoV-2

Data for the involvement of the HCWs in acute respiratory illness (ARI) management, history of exposure to any COVID-19 cases and travel history were also recorded (Table 2). Of the enrolled HCWs, 39.4% (*n* = 243) had been involved directly in the management of ARI cases. This group comprised the staff from ARI clinics, as well as the HCWs who had been involved in the treatment of ARI patients, ARI sampling and sample processing at their respective workplaces. With regard to the history of exposure to COVID-19 cases, 28.2% (*n* = 174) of the HCWs reported experiencing close or casual contact to either a confirmed case or a suspected (persons under investigation (PUIs)) COVID-19 case. As indicated by the Guidelines for COVID-19 Management in Malaysia (Ministry of Health Malaysia, 2020), PUIs are defined as those who “developed acute respiratory infection, and had traveled/resided in foreign countries, or being in close contact with confirmed cases within 14 days before onset, or attended an event associated with a known outbreak”. Close contacts are classified as those who “worked, traveled or lived together with a COVID-19 patient”. For travel history, only a small percentage of the HCWs (1%, *n* = 6) reported travel history as interstate red zone travelers or had attended a large gathering.

### 3.4. Detection of Anti-SARS-CoV-2 Nucleocapsid Total Antibodies from HCWs

All serum samples were found to be non-reactive to SARS-CoV-2 N protein (0%, *n* = 617) (Table 3). The COI readings of the tested HCWs sera ranged from 0.071 U/mL to 0.392 U/mL, which are far below the COI value of a positive reading (≥0.8 U/mL). All the serum samples from the four post-COVID-19 patients that served as additional controls were highly reactive, with the COI readings ranging from 45.56 U/mL to 157.1 U/mL. Both Elecsys^®^ reaction controls also worked well, suggesting that the performance of this assay was reliable.

With regard to this zero prevalence of SARS-CoV-2 antibodies, whether or not it has any associations with sociodemographic characteristics and potential determinants of COVID-19 exposure is unknown. However, based on the random sampling methods that were applied in this study, it can be concluded that the majority (above 78%) of the enrolled HCWs were female, young (≤40 years old) and healthy. Although this proportion is equivalent to the actual number of HCWs in Malaysia, it limits the sero-surveillance data for the opposite determinants, where the elderly group and those with underlying medical conditions are more susceptible to SARS-CoV-2 infection.

### 3.5. Survey of IPC Measures against COVID-19 among HCWs in Malaysia

COVID-19 IPC measures were assessed from previously available data from the seven studies conducted between March 2020 and February 2021 (Table 4). Bloom’s cut-off point of 80% was used for the classification of good practices towards the prevention of COVID-19. HCW participants in all the surveyed studies showed high levels of compliance (>88%) with the IPC measures [18,19,20,21,22,23]. Exceptionally, one study reported a slightly lower percentage of compliance (75%) with ”always” practicing social distancing, based on the three scales given in their questionnaires (never, occasionally and always) [24]. All studies incorporated surveillance for hand hygiene, as well as the use of face masks or PPE at public or workplaces. High compliances (≥93%) with hand hygiene were reported in these studies, which included frequent hand washing with water and soap; good hand hygiene practices before and after exposure to infected patients and contaminated areas or surfaces; and the use of alcohol-based hand-rub sanitizer. The average percentage for HCWs who practiced frequent and proper hand hygiene calculated from the seven studies was 97.5%. Wearing masks or full PPE is another important measure against COVID-19 at workplaces. At least 88% of the HCWs followed the recommendation to wear a mask or PPE as needed. High compliances with mask or PPE usage were noted from these studies, with an average percentage of 95.0%. Malaysian HCWs were also reported to have high compliance levels for practicing social distancing (92.2%), avoiding crowded places (97.6%) and traveling to high-risk areas (99.8%), performing health screenings when symptomatic (93.4%) and adherence to COVID-19 safety protocol at the workplace (98.5%).

## 4. Discussion

Population-based seroprevalence studies are important for the evaluation of population immunity against a specific infection. This study found zero prevalence of SARS-CoV-2 antibodies among asymptomatic HCWs of the USM Health Campus population in the first year of the COVID-19 pandemic. Study sampling was conducted in the middle of the serial implementation of MCOs and prior to the national COVID-19 vaccination program. During this sampling time, Malaysia was facing the third wave of COVID-19 (beginning in September 2020 and lasting until the end of March 2021), which recorded the highest peak of more than 5000 cases per day. Combating the third wave was a tougher task for the government and frontliners (including HCWs) compared to the two earlier COVID-19 waves because the sudden surge of cases overburdened healthcare facilities and staff. The Malaysian government revealed that, as of December 2020, of the 90,816 cumulative cases, a total of 1771 HCWs were positive for COVID-19 and most of them (76.7%) were infected during the third wave of the COVID-19 pandemic [28]. Hence, continuous SARS-CoV-2 serosurveys are important to estimate the COVID-19 burden and to understand the true prevalence of infections in a particular population at a specific period. The prevalence of underreported and underdiagnosed COVID-19-positive cases tends to lead to underestimations of the true number of SARS-CoV-2 infections [29].

Published data on earlier COVID-19 seroprevalence levels among Malaysian HCWs were available from several studies. A study conducted during the initial phase of the epidemic in March 2020 reported that a small proportion (4.5%) of the enrolled HCWs (*n* = 310) in Sarawak were seropositive [18]. During that time, critical shortages of PPE (gloves, medical masks, respirators, goggles, face shields, boots/shoe cover, gowns and aprons) globally had placed the HCWs and other frontliners at high risk of COVID-19 exposure. Hence, the proportion of seropositivity was found to be slightly higher in comparison to other studies conducted after the circumstances were resolved in April 2020. Another study showed similar findings of zero seroprevalence among HCWs (*n* = 400) in Klang Valley, Malaysia [16]. The sampling period was from April to May 2020, in the middle of the Malaysia’s first implementation of an MCO. The authors suggested that the role of adherence to infection control measures and appropriate PPE usage could reduce SARS-CoV-2 transmission. Additionally, a study using residual serum samples from a teaching hospital serving Kuala Lumpur and Selangor, Malaysia, also reported low seroprevalence (0.4%) based on the age-standardized population [19].

In this study, zero prevalence of antibodies against SARS-CoV-2 was recorded, although nearly half (40.0%, *n* = 246) of the enrolled HCWs had been involved directly in the management of ARI cases. ARI healthcare facilities provide diagnosis and treatment services for the management of patients presenting with respiratory symptoms, including fever, cough, runny nose, sore throat and loss of taste or smell. However, not all ARI cases are COVID-19-positive. One should bear in mind that respiratory infections can be caused by a variety of viruses (and even by bacteria or fungi), for which the clinical signs and symptoms are commonly indistinguishable from one another [30,31]. For instance, a recent study found that only 56 (28.3%) of 198 suspected COVID-19 patients in a teaching hospital in Malaysia were positive for SARS-CoV-2, while the rest had either undetected pathologies or were positive for other respiratory pathogens [32]. Another virological survey conducted in Germany reported that only 12 (0.3%) of the total 3580 samples were positive for SARS-CoV-2, whilst the highest proportions of viral pathogens detected from ambulatory patients presenting with ARI or influenza-like illness were dominated by human influenza virus A/B (23.4%), human rhinovirus (13.8%) and human metapneumovirus (5.3%) [33]. This could be one of the possibilities for the absence of seropositivity among HCWs who were involved directly in the management of ARI cases.

Zero seroprevalence indicated that the incidence of COVID-19 was low among the asymptomatic HCWs of the USM Health Campus population during the study period. One reason behind this low COVID-19 incidence could have been associated with the high compliance with IPC measures and the stringent standard operating procedures (SOPs), which were outlined by the MOH of Malaysia and specifically by the USM top management to break the COVID-19 chain [11,34]. Enforcement of IPC measures, such as the mandatory use of PPE, social distancing, hand hygiene, health screening and restriction of the number of staff and student in the campus, were practically important to prevent COVID-19 infection among the USM population and, most importantly, the community. This is supported by the Malaysian MOH statement that only 20% of the COVID-19 cases involving HCWs in Malaysia were acquired at the workplace. In contrast, 60% of cases originated in the community [11]. Additionally, the sampling period, which was during the MCOs, may have been the reason for this zero seroprevalence. The MCOs restricted many social activities. One example can be seen from the small proportion (0.5–1.0%) of the HCWs with travel history recorded in this study and in the literature survey. The effects of public health and social measures, such as implementation of MCOs, on the spread of COVID-19 can be assessed by measuring changes in case incidence and the reproduction rate at a particular time point [35]. This is useful to understand the spread of an epidemic in the context of revised pandemic preparedness and mitigation plans [36]. According to a recent study, MCO enforcement was able to reduce the time-varying reproduction rate of COVID-19 cases by up to 77.1% and 47.0% during the second and third waves, respectively [37]. The MCOs were reported to facilitate the reduction of the burden of HCWs in flattening the COVID-19 curve by decreasing the numbers of daily cases.

In addition to the serosurvey, a literature survey on IPC measures against COVID-19 was performed. Data were extracted from seven studies involving a total of 3631 respondents from the entirety Malaysia. Based on the literature, the vast majority of Malaysian HCWs responded with good practices towards each IPC measure during the pandemic (the average percentage ranged from 92.2–99.8%) [16,17,22,23,24,26,27]. Indeed, this proportion was higher than the IPC compliance among HCWs in other countries, such as Nigeria (77.6%) and Vietnam (75.8%) [38,39]. The high level of compliance with IPC measures was likely due to the mandatory requirement for all HCWs (regardless of their job position) to follow the SOPs during the COVID-19 pandemic phase [10,11]. Such surveillance data are useful for the development and implementation of policies to maintain and strengthen IPC measures in healthcare facilities, as well as communities [40]. As SARS-CoV-2 persists in circulating in the population, it is important to maintain key IPC measures, such as wearing masks and practicing proper hand hygiene, to reduce the disease transmission [34]. Other important measures during the COVID-19 third wave, in accordance with the MCO enforcement, included the implementation of a staggered return to the office and the continual enforcement of the work from home (WFH) order for administrative and academic staff; prohibition of any physical gatherings and social events; implementation of online-based meetings, classes and seminars; and daily body-temperature screening before entering the campus area. However, most of these rules were lifted as Malaysia entered the transition to the endemic phase in April 2021.

Despite the zero seroprevalence and high compliance with IPC measures among the asymptomatic HCWs, this study had several limitations. First, the sampling was conducted in the late phase of the COVID-19 pandemic. The seven-month duration of sampling corresponded to the third wave of COVID-19 phase in Malaysia, where the local cases were still lower than in the later endemic phases. The cases began to increase in late April 2021 due to the transmission of the Delta variant in the community. Second, the participants involved in this study were working merely on a voluntary basis and comprised asymptomatic individuals. The exclusion of symptomatic individuals may also have led to underestimation of the seropositivity. If the enrollment was undertaken as a compulsory screening program with all HCWs in the Hospital USM population, there might have been a chance of detecting individuals with seropositivity for SARS-CoV-2. Nonetheless, a surveillance program conducted at a teaching hospital in Malaysia reported that only 0.3% of the total HCWs (*n* = 1174) were found positive for COVID-19, although 35% of them were symptomatic [32]. Third, the sample size of this study was relatively small when compared with the total number of HCWs in Malaysia and was conducted in only one specific healthcare setting (Hospital USM). Moreover, Hospital USM is in the East Coast branch, where the COVID-19 cases were typically much lower than the central region of Peninsular Malaysia (Selangor, Kuala Lumpur and Putrajaya). Comparatively, the number of COVID-19 cases in these two regions ranged between one- and three-digit figures in the middle of December 2020 and three- and four-digit figures at the end of February 2021. This may also have contributed to the low incidence of SARS-CoV-2 seropositivity and a substantial underestimation the overall burden of SARS-CoV-2 infection among HCWs in Malaysia.

Fourth, samples were prospectively collected only from healthcare facilities because this was a cross-sectional study to assess the seroprevalence of SARS-CoV-2 among asymptomatic HCWs. Hence, there was no control group since no comparison was undertaken in accordance with the scope of the study. The primary outcome of interest was SARS-CoV-2 total antibodies seropositivity. For any HCWs with seropositivity for SARS-CoV-2, the secondary outcome of SARS-CoV-2 infection could have been combined with the participants’ self-reported data on demographics, health status and other variables for associational analysis. However, since all the sera collected from the HCWs were non-reactive, further associational analysis for the secondary outcome was not available. Fifth, this study also had a limitation in the unequal distribution of each determinant, as the majority of the participants were females, nurses and in a young age group (≤40 years old). However, in the actual population, until September 2021, nursing careers have been the predominant healthcare professions in facilities under the MOH in Malaysia [28]. Other studies also reported similar disproportionate distributions of jobs, ages, and gender categories in their sample populations among Malaysian HCWs, where nurses, young age groups and females predominate among the overall population [29,30,31,32]. This shows that the sampling groups of this study may represent the actual population of HCWs in Malaysia.

Another limitation of this study was the use of a single test that can only detect total antibodies against SARS-CoV-2 N protein. The specificity and sensitivity of the Elecsys^®^ Anti-SARS-CoV-2 immunoassay were previously reported to be 99.98% and 98.80%, respectively [41]. A validation study by Public Health England reported a specificity of 100% and a sensitivity of 83.9% for the Elecsys^®^ immunoassay [42]. Although the Elecsys^®^ Anti-SARS-CoV-2 immunoassay is highly sensitive and specific, a previous study reported that at least 20% of cases with laboratory-confirmed evidence of COVID-19 infection were negative in the three different antibody assays tested, which included the Elecsys^®^ Anti-SARS-CoV-2 immunoassay. This suggests that the use of commercial antibody assays to assess COVID-19 infection in population-based surveys is likely to substantially underestimate actual infection exposure, especially when the SARS-CoV-2 incidence in the community is high [43]. Moreover, the Elecsys^®^ immunoassay is only recommended for samples from individuals with 14 days or more post-symptom onset. As such, molecular based assays should be incorporated in future seroprevalence studies to gain knowledge on both previous and current exposure to SARS-CoV-2 positivity. Serological data in combination with molecular diagnostic tests would provide a more accurate picture of COVID-19 infection in the population. Further studies using a well-designed surveillance program for SARS-CoV-2 exposure, risk factors and IPC practices are highly recommended to achieve effective management of COVID-19 among HCWs.

## 5. Conclusions

To the best of our knowledge, this is the first and possibly the only study conducted that assesses the prevalence of SARS-CoV-2 antibodies among asymptomatic HCWs, specifically during the third wave of the COVID-19 pandemic in Malaysia. This study also provides additional information on the nationwide IPC practices against COVID-19 among Malaysian HCWs based on published data collected between March 2020 and February 2021. Data and evidence from these seroprevalence studies can be used as an indicator of population immunity, as antibodies can be detected in mild or asymptomatic cases that otherwise remain undiagnosed. Zero seroprevalence of antibodies against SARS-CoV-2 demonstrates the low incidence of COVID-19 among the asymptomatic HCWs of USM Health Campus during the pandemic phase, specifically in the middle of the third COVID-19 wave in Malaysia. Such low prevalence was also reported by other studies in Malaysia and, thus, confirms the low degree of SARS-CoV-2 transmission in hospital settings, especially among HCWs during the pre-vaccination period [23,32,34]. This low seroprevalence resembles the low proportion of immunity to COVID-19 in the population. We postulate that high compliance with IPC measures has led to low seroprevalence for SARS-CoV-2 among HCWs. However, in the absence of a COVID-19 vaccination or other control measures, the population is vulnerable to further disease spread.

## Figures and Tables

**Table 1:** Sociodemographic data for the enrolled HCWs of USM Health Campus.

Age	*n*	%
Mean (±SD) = 35.95 (8.76)		
21–40	466	75.5
41–60	148	24.0
61–70	3	0.5
Total	617	100
Gender	*n*	%
Male	126	20.4
Female	491	79.6
Total	617	100
HCW study participants	*n*	%
Nurses	397	64.3
Health attendants	129	20.9
Medical doctors	59	9.6
Assistant medical officers	10	1.6
Others	22	3.6
Total	617	100

**Table 2:** History of exposure of the HCWs to SARS-CoV-2.

Involvement in ARI Management	*n*	%
Not involved in ARI management	374	60.6
On duty at ARI clinics	94	15.2
Involved in treatment of ARI/SARI patients	76	12.3
Involved in sampling/processing samples	24	3.8
Involved in treatment and sampling/processing	49	7.9
Total	617	100.0
Exposure to COVID-19 Cases	*n*	%
No exposure to COVID-19 cases	443	71.8
Close contact with a confirmed case	50	8.1
Casual contact with a confirmed case	123	19.9
Close contact with a PUI case	1	0.2
Close contact with an international traveller	0	0
Total	617	100.0
Travel History	*n*	%
None	611	99.0
International	0	0
Interstate red zone	4	0.6
Attended large gathering	1	0.2
Attended event at any COVID-19 outbreak	0	0
Interstate red zone traveller and large gathering	1	0.2
Total	617	100.0

**Table 3:** Detection of anti-SARS-CoV-2 nucleocapsid total antibodies from HCWs (*n* = 617).

Elecsys^®^ Anti-SARS-CoV-2 from the Cobas e411 Analyzer (Roche Diagnostics)	Indication Test	*n*	Mean of COI (U/mL)	Interpretation
Serum samples from HCWs	Enrolled subjects	617	0.083	Non-reactive
Serum samples from post-COVID-19 controls	Additional controls	4	103.59	Reactive
Elecsys^®^ PC ACOV2 1	Negative control	4	0.092	Non-reactive
Elecsys^®^ PC ACOV2 2	Positive control	4	2.501	Reactive

**Table 4:** Literature survey on IPC measures against COVID-19 among HCWs in Malaysia.

IPC Practices against COVID-19 Infection	Study Setting	Duration of Study	Total Participants (*n*)	Compliance Level (%)	References
Frequent and proper hand hygiene (using water and soap or hand sanitizer)	Selangor	1–31 May 2020	193	98.4	[22]
	Perlis	29 May–27 July 2020	373	95.7 ^a^	[23]
	Selangor	18 November–18 December 2020	407	98.9 ^b^	[24]
	Klang Valley	13 April–12 May 2020	201	93.0 ^c^	[16]
	Entirety of Malaysia	30 March–6 April 2020	1719	99.6	[25]
	Kuala Lumpur	N/M	571	98.8	[26]
	Sabah	December 2020–February 2021	167	98.2	[27]
Wearing masks or PPE at public places or workplace	Serdang, Selangor	1–31 May 2020	193	96.4	[22]
	Perlis	29 May–27 July 2020	373	92.8 ^a^	[23]
	Selangor	18 November–18 December 2020	407	98.5	[24]
	Klang Valley	13 April–12 May 2020	73–198	>97.0 ^c^	[16]
	Entirety of Malaysia	30 March–6 April 2020	1719	88.8	[25]
	Kuala Lumpur	N/M	571	92.7	[26]
	Sabah	December 2020–February 2021	167	98.8	[27]
Avoiding crowded places	Serdang, Selangor	1–31 May 2020	193	99.5	[22]
	Entirety of Malaysia	30 March–6 April 2020	1719	>95.6	[25]
Practicing social distancing	Serdang, Selangor	1–31 May 2020	193	99.0	[22]
	Perlis	29 May–27 July 2020	373	94.9	[23]
	Entirety of Malaysia	30 March–6 April 2020	1719	99.7	[25]
	Sabah	December 2020–February 2021	167	75.0	[27]
Avoiding travelling to high-risk areas	Serdang, Selangor	1–31 May 2020	193	100.0	[22]
	Entirety of Malaysia	30 March–6 April 2020	1719	99.5	[25]
Health screening or screening-seeking behaviour if symptomatic	Serdang, Selangor	1–31 May 2020	193	90.7	[22]
	Entirety of Malaysia	30 March–6 April 2020	1719	96.0	[25]
Adherence to workplace COVID-19 safety protocol	Selangor	18 November–18 December 2020	407	98.5	[24]

^a^ Recommended precautionary practices for hand hygiene were classified as “often” and “always”; ^b^ data were based on the average number of HCW respondents who practiced hand hygiene both before and after contact with each patient; ^c^ adherence to PPE measures with different types of exposure at workplace; N/M: not mentioned in the study.

## Data Availability

All data relevant to this review are included in the text and references.
